# Correspondents

**Published:** 1853-09

**Authors:** 


From the Boston Med'cal and Surgical Journal.

*To the Editor of the Boston Medical and Surgical Journal.*

SIR:—As the lecture season is about closing, I will devote an hour to give you a few medical items.

The Lectures of the Faculty of Medicine commence the first of November, and continue till the second week in August; although private instruction continues during the whole year. As the Faculty is composed of twenty-six professors, of course there are various changes among the lecturers during so long a session. One very interesting feature in the medical school in Paris, is the system of "*concours;*" some account of which may not be devoid of interest to your numerous readers. Having had a fair opportunity, during the past season, to witness how this system is conducted, I will give you a brief acccount of it. Formerly the professors obtained their appointment by *concours*; now they are appointed by the *Emperor*, but all places below them are determined by this test—such as *agreges*, *internes* of the hospital, &c. Agreges are under-professors—*corps reserve*—kept to fill the places of the professors, if absent or sick; or, in case of death or resignation, to supply their places permanently. During the past season, there have been *concours* in medicine, surgery, anatomy, obstetrics and chemistry. The qualifications of a candidate to be an agrege, are; 1st, that he must be French by birth; 2nd, 25 years of age; 3rd, a graduate as doctor in medicine. The term of *agregation* is nine years, and the trials take place every three and six years; consequently only one half of the places are filled at each concours.—That is in medicine there are ten agreges, and five are chosen at each trial; in surgery six, and three are chosen; and so on in other departments.

As a specimen of the trials, I will select those of medicine and surgery as fair examples of others. In the first place, a series of subjects in medicine, surgery or physiology is selected by the Faculty, and given to the competitors by lot, who have five hours allowed them to prepare themselves, and who are obliged to speak extemporaneously. Second, subjects in mediciue or surgery are given out, upon which candidates are allowed three hours time for preparation, in the presence of one of the judges, with closed doors and without books. Each candidate has forty-five minutes to speak upon his subject at his trial. Third, subjects, medicine or surgery; term of speaking the same, with twenty-four hours' preparation at their own domicile. Fourth, lessons clinique; time the same. Two patients are selected in the hospital, and each candidate is allowed twenty minutes to examine them; then he has to give his diagnosis, &c., before the judges. Fifth, each candidate receives a subject, upon which he must write and have finished a *thesis* within twelve days (and some of them comprise one hundred and sixty pages ) This must be defended against the attacks of two of the competitors—each being allowed thirty minutes.—Hence each candidate defends his thesis during one hour, and has the opportunity of making two attacks upon his fellow competitors; in the latter consideration he has three days' grace to prepare himself for the onslaught. The judges are seven in number—composed of five professors of the Faculty, and two agreges. The latter have no voice in the final vote for the successful candidate, unless some one of the former is deprived of being present by sickness or some other unavoidable detention.

The test of the concours is truly a severe one and well calculated to measure swords intellectually. For instance, that of medicine, last winter, commenced with over thirty competitors; yet before it was terminated, the number diminished to fourteen, out of which only five could be chosen. The trials are all public; hence, at times, as in the race, the feeling "outside" is intense, as each candidate, in the estimation of his friends, wins, or falls with the prize almost within his grasp. At some of the sittings I have seen twelve hundred persons present. If a man fails, he has but to try again, when the next concour comes around. It is said that even M. Velpeau failed twice—but, nothing daunted, succeeded at last.

Perhaps it may be said that this system is open to some objections—that those more ready of speech may sometimes appear to greater advantage than those less fluent, yet at the same time more profound in intellect. However this may be, it is certainly superior to any system where "preferment goes by favor." From this hasty sketch you will observe upon what basis these trials are conducted. It was only three or four years since such men as Nelaton and Malgaigne were concouring for professorships. Now they sit in judgment upon others.

In your Journal of May 25, I notice some remarks from the New York Medical Gazette upon the experiments of per-chloride of Iron in aneurism, by Dr. Pravas of Lyons. Not long since I saw M. Velpeau, at La Charite, make trial of the remedy in an aneurism upon the left arm of a young man. He injected a few drops, and it coagulated the blood in the part so that the tumor felt somewhat hard. In six or seven days he repeated the experiment; but it did not finally succeed. He then ligated the humoral artery.—M. Lenois, at Hopital Neckar, has used it once, but without much better success. However, when other cases present, it will be tried again.

M. Bennet, of Lyons, has recently addressed a note to M. Lallemand, upon the employment of the paste of the chloride of zinc in aneurism. He gives a case where he has been using it upon an aneurism in the subclavicular region. It acts as a gradual cautery, distroying and healing, so that several small arteries have been acted upon without producing any hemorrhage. The tumor is nearly consumed, and all seems to promise well. The final result will be given. The idea of using caustics in such cases is not new, but M. B. claims the use of zinc as such.

M. Nelaton, at l'Hopital des Cliniques de la Faculte de Medecine, operated, one week ago to-day, for stone: the patient a man. He made the recto-vesical operation—with this exception, that instead of cutting into the bladder behind the prostrate, as is usually done in this method, he cut through the prostrate itself. As he had diagnosed a very large calculus and several small ones—the latter lying in the prostatic portion of the uretha—he decided on this somewhat novel method. After the patient had been subjected to the influence of chloroform, the anus was readily dilated so as to remain lax, by the fingers, and the operation proceeded in the usual manner. The *lithotome* was used to enter the bladder: in fact, it is preferred by the French surgeons. The largest calculus was nearly round, measuring an inch and a half in diameter.—Four smaller ones were also removed, but with little hemorrhage. The anus was not wounded. At this date, patient is doing well. The great dread in rectal operations is a permanent fistula.

M. Velpeau has been in the habit, for a long time, of using a solution of sulphate of iron, in erysipelas; and several other surgeons here think much of the collodion. M. Aran, of late, has had the idea of combining the two, in proportions equal by weight. He thinks that the medicinal properties are thus increased, and that combined, they give a better security to the inflamed skin, from the air and other agents.

Last week, at the Academy of Medicine, a paper was offered, taking the ground that the introduction of vaccine virus had been the cause of typhoid fever. The Academy quietly disposed of it—thinking, perhaps that the author's brain needed repose.

Cholera is raging in the East to some extent. At St. Petersburg July 18, there were 633 patients under treatment, 79 new cases, 32 deaths, and 35 cures. From the intense heat and the absence of rains, the deaths at Calcutta, recently, were 1,100 in two days.

But my hour has expired, and I fear I have already written too much.

Respectfully, A. B. H.

PARIS, August 1. 1853.

